# Circular RNA hsa_circ_0004277 Stimulates Malignant Phenotype of Hepatocellular Carcinoma and Epithelial-Mesenchymal Transition of Peripheral Cells

**DOI:** 10.3389/fcell.2020.585565

**Published:** 2021-01-12

**Authors:** Chuanrong Zhu, Yang Su, Lei Liu, Shaochuang Wang, Yuting Liu, Jinsheng Wu

**Affiliations:** ^1^Department of Hepatobiliary and Pancreatic Surgery, The Affiliated Huaian No.1 People's Hospital of Nanjing Medical University, Huai'an, China; ^2^Department of Surgery, The Affiliated Huaian No.1 People's Hospital of Nanjing Medical University, Huai'an, China

**Keywords:** exosomes, EMT, circRNA, hepatocellular carcinoma, ZO-1

## Abstract

Accumulating evidence shows that exosomal circRNAs reflect the physiological status of donor cells, and various cell reactions are induced after exosomal circRNAs are captured by recipient cells. In this study, qRT-PCR was performed to detect circ-0004277 expression in hepatocellular carcinoma (HCC) cell lines, tissues, and plasma exosomes. The effects of circ-0004277 on the proliferation and migration of HCC cells were assessed by cell counting, 5-ethynyl-2′-deoxyuridine assays, Transwell migration assays, and tumor formation in nude mice. We found that circ-0004277 was significantly upregulated in HCC cells, tissues, and plasma exosomes compared to that in normal controls. Overexpression of circ-0004277 enhanced the proliferation, migration, and epithelial-mesenchymal transition (EMT) of HCC cells *in vivo* and *in vitro*. Furthermore, exosomes from HCC cells enhanced circ-0004277 expression in surrounding normal cells and stimulated EMT progression. ZO-1, a tight junction adapter protein, was downregulated in HCC tissues. In conclusion, our findings suggest that circ-0004277 promotes the malignant phenotype of HCC cells via inhibition of *ZO-1* and promotion of EMT progression. In addition, exosomal circ-0004277 from HCC cells stimulates EMT of peripheral cells through cellular communication to further promote the invasion of HCC into normal surrounding tissues.

## Introduction

Hepatocellular carcinoma (HCC) is the most prevalent primary liver cancer (Kim et al., 2017) and the second most common cause of cancer-related deaths. It is the sixth most prevalent cancer worldwide, with poor prognosis and high malignancy (Bosetti et al., 2009; Forner et al., 2012; Niendorf et al., 2015). Factors associated with HCC development include viruses, chemical substances, and congenital and acquired metabolic diseases (Carr and Guerra, 2017). Although there are multiple therapeutic options for HCC, the prognosis of HCC remains poor, with a 5-year survival rate of <20% as a result of high metastasis and recurrence rates (Bruix et al., 2011). Therefore, further studies on the pathogenesis of HCC are important.

Circular RNA (circRNA) was first discovered in 1976 (Eger et al., 2018). However, owing to limited research techniques, it was considered meaningless until it became a significant research topic in recent years. CircRNAs are covalently closed single-stranded RNA molecules from exons via alternative mRNA splicing (Jin et al., 2016). CircRNAs, as conserved and stable non-coding RNAs in mammalian cells, are known to be involved in various cancer-related processes (Zhao et al., 2017), particularly in colon (Hsiao et al., 2017), bladder (Zhong et al., 2017), gastric (Tian et al., 2018), and non-small cell lung cancers (Yao et al., 2017). Many studies have demonstrated that circRNAs are closely associated with HCC. A study by Han et al. has shown that circular RNA circMTO1 serves as a sponge of microRNA-9 to inhibit the progression of hepatocellular carcinoma (Han et al., 2017). It has also been proven that circRNAs may participate in the pathogenesis of HCC, such as circRNA-100338 (Huang X. Y. et al., 2017), circ_0005075 (Shang et al., 2016), circular RNA ciRS-7 (Cdr1as) (Xu et al., 2017; Su et al., 2019), and circ-ZEB1.33 (Gong et al., 2018). Therefore, it is important to explore the function of circRNAs in the occurrence and progression of HCC.

Exosomes are small vesicular bodies present in various cells and contain mRNAs, miRNAs, and proteins that may regulate signaling pathways in recipient cells (Zhu et al., 2017). In addition, proteins and nucleic acids are delivered by exosomes to regulate intercellular communication between stromal cells and cancer cells (Pan et al., 2017). According to Steinbichler et al., metastasis is a complex multi-stage process, including the migration of cancer cells, survival in blood vessels, and adhesion to and colonization of host organs. As exosomes can be targeted by oncological therapy and affect each stage of this cascade (Steinbichler et al., 2017), there is reason to believe that exosomes may be involved in the occurrence and development of HCC.

Recent research shows that Circular RNA IARS secreted by pancreatic cancer cells and located within exosomes regulates endothelial monolayer permeability to promote tumor metastasis (Li J. et al., 2018). Tight junctions are important for maintaining the mechanical strength and permeability of the mucosal epithelium (Basler et al., 2016), which regulates the movement of ions and macromolecules between endothelial and epithelial cells (Lochhead et al., 2020). The tight junction protein ZO-1 is one important component that not only regulates cell material transport and maintains epithelial polarity, but also plays an important role in cell proliferation and differentiation and tumor cell invasion (Hsu et al., 2017; Haas et al., 2020).

Here, we hypotheses that circ-0004277 promotes the malignant phenotype of HCC cells via inhibition of *ZO-1* and promotion of EMT progression. In addition, exosomal circ-0004277 from HCC cells stimulates EMT of peripheral cells through cellular communication to further promote the invasion of HCC into normal surrounding tissues. In this study, qRT-PCR was utilized to detect the expression of six well-known tumor-related circRNAs in the human-derived liver cell line HL-7702 and HCC cell lines. The results showed that only circ-0004277 expression was increased in HCC cell lines. We verified this result in a population-based study. Subsequently, *in vitro* and *in vivo* assays were conducted to detect the role of circ-0004277 in cell proliferation and migration, and the results showed that circ-0004277 promoted the malignant phenotype of HCC. However, there are no data on the biological role of circ-0004277 in HCC. The present study was performed to investigate whether circ-0004277 contributed to the progression of HCC and to elucidate the underlying mechanisms.

## Materials and Methods

### Study Subjects and Design

All the subjects provided written informed consent, and the study protocol was approved by the Ethics Committee of the Affiliated Huaian No.1 People's Hospital of Nanjing Medical University. Plasma specimens from 60 HCC patients and 60 negative controls were analyzed, along with 60 matched tumor and paired adjacent normal tissues from HCC patients from The Affiliated Huaian No.1 People's Hospital of Nanjing Medical University.

### Cell Transfection and Cultures

The Shanghai Cell Bank of the Chinese Academy of Sciences provided normal human hepatic cells (HL-7702 cells) and the human HCC cell lines HepG2, Bel-7402, MHCC97, Huh-7, and SMMC-7721. Cell culture was performed using RPMI 1640 culture medium containing 10% inactivated newborn bovine serum, 100 U/mL streptomycin, and 100 U/mL penicillin at 37°C under 5% CO_2_. The medium was replaced at an interval of 2–3 d. Passage was performed when the cell confluency reached 90% to maintain logarithmic cell growth. The assays were conducted using cells in the logarithmic growth phase. Lentiviruses containing overexpressing sequences or small hairpin RNA (shRNA) were obtained from GenePharma (Shanghai, China). All transfection experiments were performed by following the manufacturer's instructions using Lipofectamine 2000 reagent (Invitrogen, Carlsbad, CA, USA). One shRNA targeting the backsplice sequence of circ-0004277 was designed. In brief, shRNA or scrambled sequences were cloned into the GenePharma Supersilencing Vector. For Lentivirus shRNA vector production, vectors were cotransfected with the Helper vector-I in the 293T packaging cell line. To recapitulate circRNA, the genomic sequence for circ-0004277 was amplified, and then the sequence was inserted into pcDNA3.0 vector. Stably transfected cells were selected via treatment with 2 μg/mL puromycin for 2 weeks. Detailed sequences were depicted in Table 1.

**Table 1:** Sequences of primers for qRT-PCR.

**Name**		**Sequence**
circ-0000284	Forward	5′-TATGTTGGTGGATCCTGTTCGGCA-3′
	Reverse	5′-TGGTGGGTAGACCAAGACTTGTGA-3′
circ-0004277	Forward	5′-AACAAAAGCCAGTCACAGCA-3′
	Reverse	5′-CATCAATCGCTTGTCCTTCA-3′
circ-Cdr1as	Forward	5′-TCAACTGGCTCAATATCCATGTC-3′
	Reverse	5′-ACCTTGACACAGGTGCCAT-3′
circ-Foxo3	Forward	5′-GGGGAACTTCACTGGTGCTA-3′
	Reverse	5′-TCTTGCCAGTTCCCTCATTC-3′
circ-SHPRH	Forward	5′-AGGCAATGCTGAAAACTGCT-3′
	Reverse	5′-GCCACGTTGAGAAAACGAGT-3′
circ-ITCH	Forward	5′-TGATCCTCTTGGTCCATTGC-3′
	Reverse	5′-AGCCCATCAAGACAAATTGA-3′
E-cadherin	Forward	5′-CGAGAGCTACACGTTCACGG-3′
	Reverse	5′-GGGTGTCGAGGGAAAAATAGG-3′
ZO-1	Forward	5′-CAACATACAGTGACGCTTCACA-3′
	Reverse	5′-CACTATTGACGTTTCCCCACTC-3′
N-cadherin	Forward	5′-TCAGGCGTCTGTAGAGGCTT-3′
	Reverse	5′-ATGCACATCCTTCGATAAGACTG-3′
ZEB-1	Forward	5′-GATGATGAATGCGAGTCAGATGC-3′
	Reverse	5′-ACAGCAGTGTCTTGTTGTTGT-3′
GAPDH	Forward	5′-GCACCGTCAAGGCTGAGAAC-3′
	Reverse	5′-GGATCTCGCTCCTGGAAGATG-3′
circ_0004277 shRNA	Forward	5′-GATCCCCCTTCCACCAGCAAGTACGTCTTCCTGTCAACGTACTTGCTGGTGGAAGTTTTTGGAAA-3′
	Reverse	5′-AGCTTTTCCAAAAACTTCCACCAGCAAGTACGTTGACAGGAAGACGTACTTGCTGGTGGAAGGGG-3′

### RNA Isolation and qRT-PCR

TRIzol reagent (Life Technologies, Carlsbad, CA, USA) was used to extract cellular RNA. We measured the RNA concentration before storage at −80°C for future analysis using an ABI 7900 Fast Real-Time PCR System (Applied Biosystems). Powerup SYBR Green qPCR master mix was used for real-time PCR, which was performed in triplicate. The polymerase chain reaction was conducted as follows: an initial denaturation for 10 min at 95°C followed by 40 cycles at 95°C for 15 s and 1 min of annealing/extension at 60°C. A previously described method was used to determine and normalize the RNA expression levels to GAPDH. The PCR primers used are listed in Table 1.

### Sanger Sequence

To confirm that circ-0004277 sequence amplified by primers was identical to that in circbase and encompassed the circular 3′/5′-end, we performed Sanger sequence. Sanger sequencing was performed by inserting amplified products into the T vector. After determining the length, the back-splice joint of circ-0004277 was analyzed by constructing different primers. We authorized Invitrogen (Shanghai, China) to construct the primers and Realgene (Nanjing, China) to perform Sanger sequencing.

### RNase R Digestion

To verify the circRNA nature of circ-0004277, we performed RNase R digestion assay. Total RNA (5 μg) was cultured with 3U/μg of RNase R (Epicenter Biotechnologies, Shanghai, China) for 15 min at 37°C. The RNase R digestion was performed in triplicate.

### Cell Migration Assay

Cell migration assay was performed as previously described (Ma et al., 2016). In brief, 5 × 10^4^-10^5^ cells in serum-free media were seeded into the upper layer of a Transwell membrane insert (pore size: 8 μm) in a 24-well plate (Corning). Subsequently, medium containing 10% fetal bovine serum was used as an attractant and added to the bottom chamber. HCC cells were fixed in methanol and stained with crystal violet 36 h later. HL-7702 cells were subjected to fixation in methanol and to crystal violet staining 96 h later. Image-Pro Plus 6.0 was used for cell counting.

A wound healing assay was performed to measure cell migration. The cells were seeded in 6-well plates and scratched with a 10 μL pipette tip. After washing three times, the non-adherent cells were removed. Cell migration toward the wound 12 h post-scratching was photographed using an inverted microscope (Olympus, Tokyo, Japan). The wound areas were analyzed using Image J2x (Rawak Software Inc., Stuttgart, Germany) to assess cell migration capacity.

### Cell Proliferation Assay

The Cell Counting Kit-8 (CCK-8) (Beyotime Institute of Biotechnology, Nantong, China) was used to detect HCC cell proliferation. Briefly, before adding CCK-8, cells were added to a 96-well plate at 1,000 cells per well. The absorbance was measured at 450 nm. Cells in each group were tested five times, while the experiments were conducted in triplicate.

The 5-Ethynyl-2′-deoxyuridine (EDU) incorporation assay was conducted as per the manufacturer's instructions using an EDU detection kit (Keygen, Nanjing, China).

### Tumorigenicity Assay

Nude mice, aged 4–5 weeks, were obtained from the Shanghai Institute for Biological Sciences and maintained under sterile specific pathogen-free conditions. The nude mice were injected subcutaneously with 1 × 10^6^ SMMC-7721 cells containing stably overexpressed circ-0004277 or corresponding blank vectors to determine tumor growth and metastasis. The size of the bearing tumor was measured every week and harvested by sacrifice of mice 4 weeks after seeding. The subcutaneous formatted tumor nodes were collected for subsequent detection. The study was approved by the Institute Research Medical Ethics Committee of The Affiliated Huaian No.1 People's Hospital of Nanjing Medical University and conducted under the Guide for the Care and Use of Laboratory Animals of the National Institutes of Health.

### Metastasis Assays

The treated SMMC-7721 cells were injected into nude mice via the tail vein. The mice were evaluated after 28 days. Lung metastatic nodules were counted to determine the metastasis of cells subjected to different treatments. Hematoxylin and eosin (HE) staining was used to confirm the metastatic tumors.

### Immunohistochemical Staining

As previously indicated, immunohistochemistry analysis was performed by immunohistochemical staining (Henri et al., 2016). Primary antibodies against Ki-67, N-cadherin, and ZO-1 were obtained from Abcam (Cambridge, MA, USA). A fluorescence microscope (DP80, Olympus) was used for staining analysis.

### HE Staining

Tissues were fixed in 10% formalin, processed, and embedded in paraffin. For morphological analysis, multiple 10-μm-thick sections were prepared and stained with hematoxylin and eosin.

### Exosome Isolation

To remove cells and cell debris, the culture medium was collected and centrifuged for 15 min at 3,000 × g. A 0.22 μm polyvinylidene difluoride (PVDF) filter (Millipore) was used to filter the supernatant. Subsequently, a moderate ExoQuick exosome precipitation solution (System Biosciences) was added to the filtered culture media. After 24 h of refrigeration, the ExoQuick/biofluid mixture was centrifuged for 30 min at 1,500 × g, and the supernatant was removed. The exosomes appeared as beige or white pellets at the bottom of the vessel.

### Transmission Electron Microscopy (TEM)

Before TEM analysis, exosomes were suspended in 100 μL phosphate buffered saline (PBS), fixed in 5% glutaraldehyde at incubation temperature, and then kept at 4°C. Based on the TEM sample preparation procedures, a drop of exosome was added to a carbon-coated copper grid and immersed in 2% phosphotungstic acid solution (pH 7.0) for 30 s. A TEM (Tecnai G2 Spirit Bio TWIN, FEI, USA) was used to observe the preparations.

### Exosome Labeling

Exosomes were isolated from 1.5 × 10^6^ HCC cells and suspended in 100 μL PBS containing 1 mL of mixed PKH67 (Sigma, St. Louis, MO, USA, in Diluent C). After incubation for 4 min at ambient temperature, exosome labeling was terminated by adding 2 mL of 0.5% BSA, and Exoquick exosome precipitation solution was added to isolate the dyed exosomes. After suspending the exosomes into 9.6 mL basal medium, 250 μL medium was added to the sub-confluent layer of HL-7702 cells. Cells were washed and fixed at room temperature after 3 h of incubation at 37°C. For nuclei staining, 4,6-diamidino-2-phenylindole (DAPI, Sigma) was added for 10 min. The stained cells were observed under a fluorescence microscope (Zeiss, LSM700B, Germany).

### Nanoparticle Tracking Analysis (NTA)

We measured the exosome particle size and concentration using NTA with ZetaView PMX 110 (Particle Metrix, Meerbusch, Germany) and corresponding software ZetaView 8.04.02. Isolated exosome samples were appropriately diluted using 1X PBS buffer (Biological Industries, Israel) to measure the particle size and concentration. NTA measurement was recorded and analyzed at 11 positions. The ZetaView system was calibrated using 110 nm polystyrene particles. Temperature was maintained around 23°C and 37°C.

### RNA Binding Protein Immunoprecipitation (RIP) Assay

This assay was performed in strict accordance with the procedures of the Magna RIPTM RIP Kit (Millipore, Billerica, MA, USA). After cell lysis, antibodies to be detected were added at a working concentration of 8 μg per reaction system. After incubation overnight in a shaker at 4°C, it was reheated to room temperature for 1 h. Then, protein G magnetic beads were added to capture the complexes. After rinsing with buffer, the RNA was extracted. Relative expression of circ-0004277 and *ZO-1* were quantified by qRT-PCR.

### Western Blot

The isolation and qualification of total proteins was performed using radio immunoprecipitation assay lysis buffer (Sigma) and a BCA detection kit (Keygen, Nanjing, China), respectively, as instructed by the manufacturer. Sodium dodecyl sulfate-polyacrylamide gel electrophoresis was used to separate equivalent amounts of protein before being transferred to a PVDF membrane. Primary antibodies were applied as follows: rabbit anti-human IgG antibodies against ZEB-1(1:500, #3396) (Cell Signaling Technology, Beverly, MA, USA), β-actin (1:500, ab8227), TSG101 (1:1000, ab125011), CD63 (1:1000, ab217345), N-cadherin (1:500, ab18203), ZO-1 (1:500, ab96587), and E-cadherin (1:500, ab11512) (Abcam). Image J software (Rawak Software Inc., Stuttgart, Germany) was used for data analysis. All experiments were repeated independently in triplicate.

### Immunofluorescence (IF)

Cells were fixed in 4% paraformaldehyde, sealed with Immnol Fluorence Staining Secondary Antibody Dilution Buffer (Beyotime), and then incubated with a 1:200 dilution of ZO-1 antibody (ab96587, Abcam) at 4°C for 24 h. After washing, cells were incubated in a 1:200 dilution of FITC-labeled goat anti-rabbit IgG (H+L) (Beyotime) for 30 min at 37°C. DAPI was prepared for nuclei staining at a 1:1000 dilution for 5 min. Images were captured with confocal laser scanning microscope (Carl Zeiss, Jena, Germany). Cell fluorescence was analyzed by Image J software (Rawak Software, Inc. Germany).

### Statistical Analysis

The characteristic differences between HCC patients and negative controls were assessed using a two-sided χ^2^-test. The paired *t*-test was performed to detect the differential expression of circ-0004277 and *ZO-1* in cancer tissues compared with adjacent non-malignant tissues. The unpaired Student's *t*-test was performed to assess the significance of other between-group differences. The AUC values for plasma exosomal circ-0004277 were reflected in the receiver operating characteristic (ROC) curve. The optimal cutoff value of circ-0004277 was determined by the maximal Youden index. All statistical analyses were conducted using SPSS 22.0 (IBM, SPSS, Chicago, IL, USA) and GraphPad Prism V5.0 (GraphPad Software, Inc., La Jolla, CA, USA). A two-sided *P* < 0.05 indicated statistical differences.

## Results

### Characteristics and Expression of Circ-0004277 in HCC

**Figure 1:**
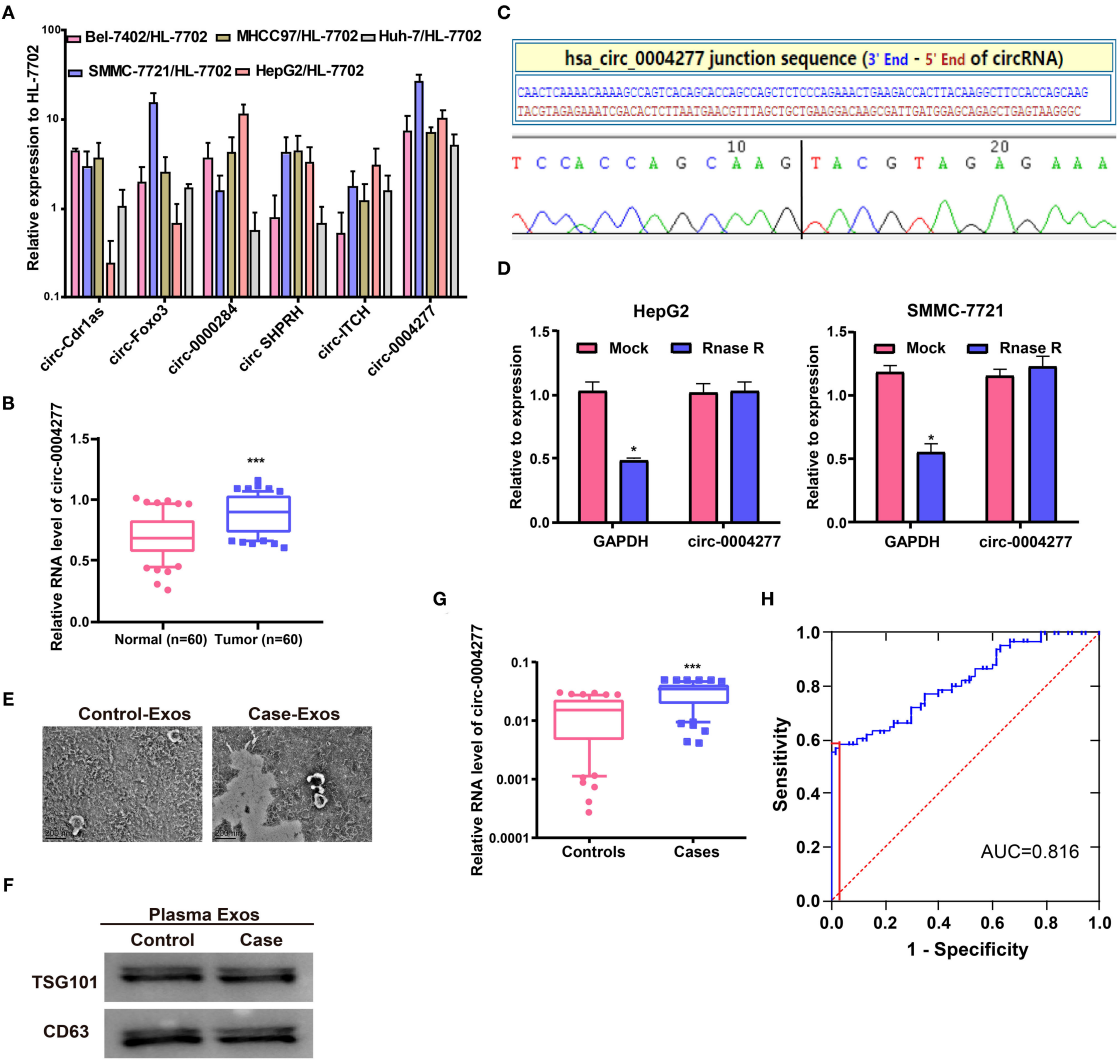
Characteristics and expression of circ-0004277 in HCC. **(A)** The expression level of six well-known circRNAs was detected in the normal human hepatic cell line HL-7702 and HCC cell lines through qRT-PCR. **(B)** qRT-PCR detection of the relative expression of circ-0004277 in paired HCC tumor and normal tissues (*n* = 60). **(C)** The sequence of circ-0004277 in circBase (upper panel) showed consistent results with Sanger sequencing (lower panel). **(D)** Circ-0004277 was resistant to RNaseR digestion in HCC cell lines. **(E)** Micrographs of plasma-derived exosomes in negative controls (left) and HCC patients (right, bars = 200 nm). **(F)** Western blots of CD63 and TSG101 in circulating exosomes. **(G)** qRT-PCR of circ-0004277 in plasma-derived exosomes. **(H)** ROC curve of exosomal circ-0004277 signature. Results are reported as mean ± SD. *P < 0.05, ***P < 0.001. All experiments were performed in triplicate.

qRT-PCR was carried out to detect differences in the expression of six well-known circRNAs in cancer. The results showed that only circ-0004277 was stably increased in the six HCC cell lines compared to that in HL-7702 cells, indicating that circ-0004277 may be involved in the occurrence of HCC (Figure 1A). Furthermore, analysis of circ-0004277 levels in HCC tumors and paired adjacent normal tissues showed significantly higher circ-0004277 expression in HCC tissues (Figure 1B). The characteristics of HCC patients and negative controls are shown in Supplementary Table 1. As shown in the Figure 1C, we performed Sanger sequence to confirm that circ-0004277 sequence amplified by primers was identical to that in circbase and encompassed the circular 3′/5′-end. RNaseR has no influence on circRNA and dissolves linear RNA characterized by a free 3′end (Wang et al., 2019). RNaseR was added to total RNA to further confirm the nature of circ-0004277. The assay demonstrated that circ-0004277 was indeed a circRNA and resistant to RNase R digestion (Figure 1D). These results showed that our primers are specific and the production of the qRT-PCR is indeed circ-0004277.

Exosomal RNAs were extracted from plasma. Plasma-derived exosomes from HCC patients and controls were both isolated and characterized. The size of exosomes from HCC patients was similar to that of the controls, as indicated by TEM (Figure 1E). Western blot analysis showed the presence of TSG101 and CD63 as exosome biomarkers (Figure 1F). Circ-0004277 expression was then detected in exosomes from plasma. HCC patients showed significantly higher circ-0004277 expression in exosomes than negative controls (*P* < 0.05, Figure 1G). Moreover, ROC curves were used to evaluate the potential role of exosomal circ-0004277 as a biomarker. The effect of exosomal circ-0004277 on diagnosing HCC is presented in Figure 1H. The under area of ROC curve was 0.816 (95% CI: 0.741 – 0.891). The Youden index was 0.550 and the sensitivity and specificity were 58.3 and 96.7% respectively. These results suggest that exosomal circ-0004277 might be a useful marker for distinguishing HCC patients.

### Circ-0004277 Promotes HCC Cell Proliferation

**Figure 2:**
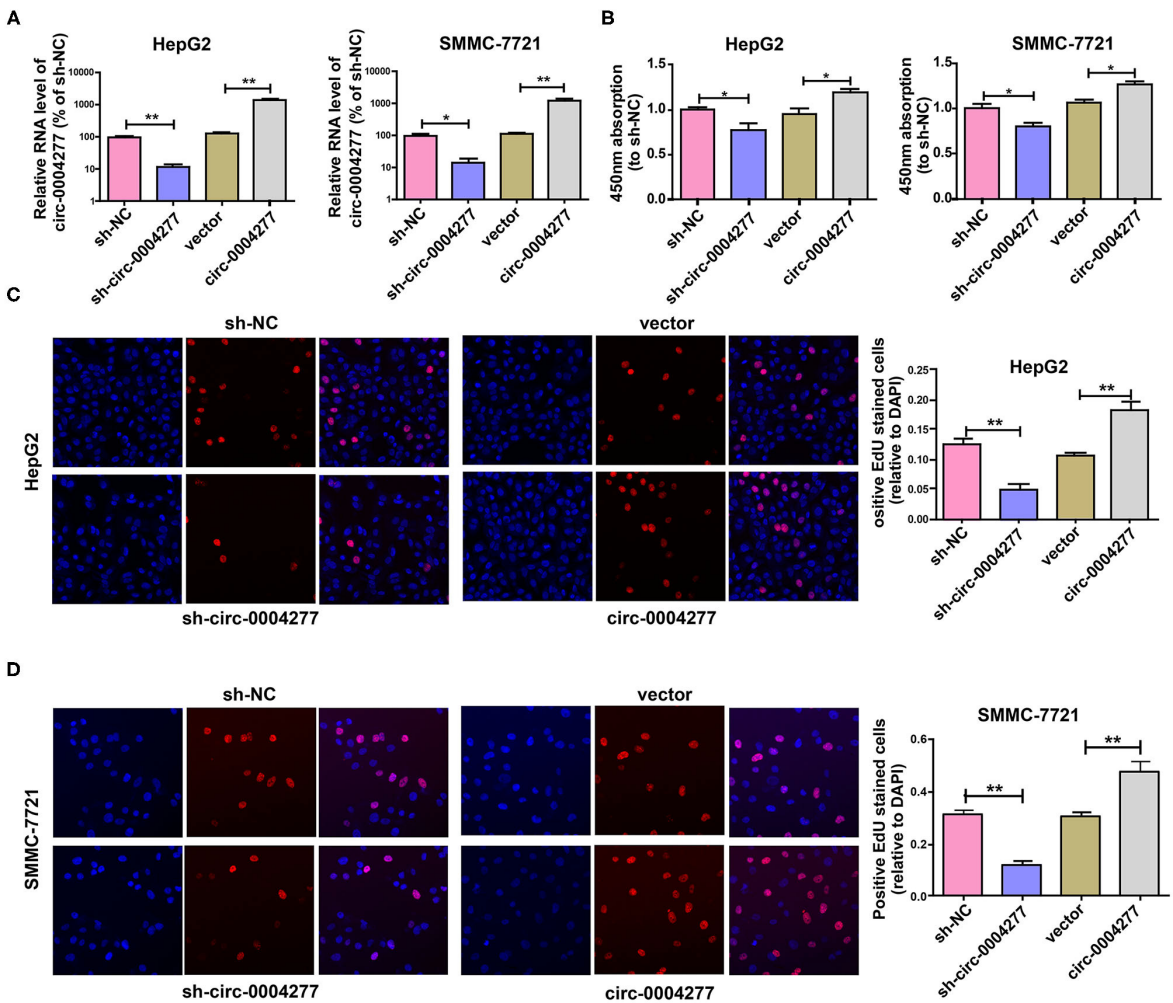
Overexpressed circ-0004277 promotes HCC cell proliferation. Circ-0004277/NC shRNA lentiviral vector was transfected into HCC cells, named sh-circ-0004277 and sh-NC, respectively. Circ-0004277/NC lentiviral vector was transfected into HCC cells, named circ-0004277 and vector, respectively. **(A)** After transfection, RNA levels of circ-0004277 were detected by qRT-PCR. **(B)** Influence of circ-0004277 lentiviral and shRNA lentiviral transfection on the proliferation of human HCC cells through CCK8 assay. **(C)** Influence of circ-0004277 lentiviral and shRNA lentiviral transfection on the proliferation of human HepG2 cells through EdU assay. **(D)** Influence of circ-0004277 lentiviral and shRNA lentiviral transfection on the proliferation of human SMMC-7721 cells through EdU assay. Results are reported as mean ± SD. *P < 0.05, **P < 0.01. All experiments were performed in triplicate.

To explore the biological role of circ-0004277 in HCC, circ-0004277 expression was silenced by transfection with a circ-0004277 shRNA lentiviral vector. In addition, circ-0004277 expression was also increased by transfection with a circ-0004277 lentiviral vector. The results showed that transfection with circ-0004277 shRNA inhibited circ-0004277 expression in HCC cells, whereas transfection with the circ-0004277 lentiviral vector upregulated circ-0004277 levels in HCC cells (Figure 2A). As our primers are specific and the production of the qRT-PCR is indeed circ-0004277. Thus, we believe that the overexpression of circ-0004277 generates the circRNA circ-0004277. Meanwhile, we found that circ-0004277 overexpression or downregulation didn't modulate the expression its host gene WDR37 (Supplementary Figure 1). To explore the influence of circ-0004277 on cell proliferation, CCK-8 and EdU experiments were conducted. The results of the CCK-8 experiment revealed that downregulated circ-0004277 significantly reduced the proliferation of HCC cells. In contrast, circ-0004277 overexpression promoted HCC cell proliferation (Figure 2B). The results of the EdU experiment was identical to those of CCK-8 (Figures 2C,D). In conclusion, these results indicate that circ-0004277 positively regulates HCC cell proliferation.

### Circ-0004277 Promotes HCC Cell Migration

**Figure 3:**
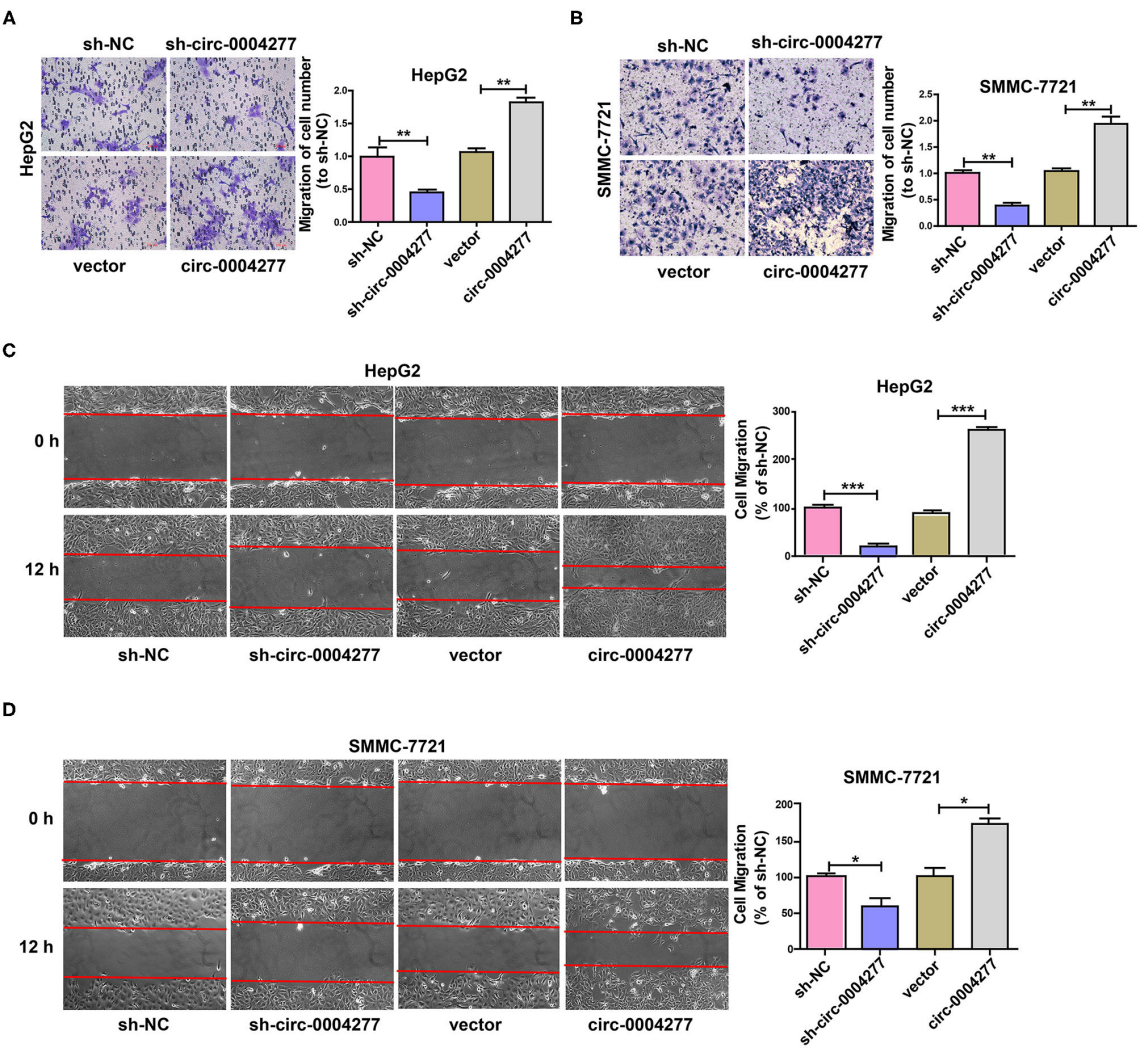
Overexpressed circ-0004277 promotes HCC cell migration. Circ-0004277/NC shRNA lentiviral vector was transfected into HCC cells, named sh-circ-0004277 and sh-NC, respectively. Circ-0004277/ NC lentiviral vector was transfected into HCC cells, named circ-0004277 and vector, respectively. **(A)** Influence of circ-0004277 lentiviral and shRNA lentiviral transfection on the migration of human HepG2 cells through Transwell migration assay. **(B)** Influence of circ-0004277 lentiviral and shRNA lentiviral transfection on the migration of human SMMC-7721 cells through Transwell migration assay. **(C)** Influence of circ-0004277 lentiviral and shRNA lentiviral transfection on the migration of human HepG2 cells through wound healing assay. **(D)** Influence of circ-0004277 lentiviral and shRNA lentiviral transfection on the migration of human SMMC-7721 cells through wound healing assay. Results are reported as mean ± SD. *P < 0.05, **P < 0.01, ***P < 0.001. All experiments were performed in triplicate.

To explore the influence of circ-0004277 on cell migration, a Transwell migration assay was conducted and showed that downregulated circ-0004277 significantly decreased the migration of HCC cells. Conversely, overexpression of circ-0004277 significantly promoted HCC cell migration (Figures 3A,B). The wound healing assay showed similar results (Figures 3C,D). These results indicate that circ-0004277 has a positive regulatory effect on HCC cell migration.

### Circ-0004277 Promotes EMT Progression and Inhibits ZO-1

**Figure 4:**
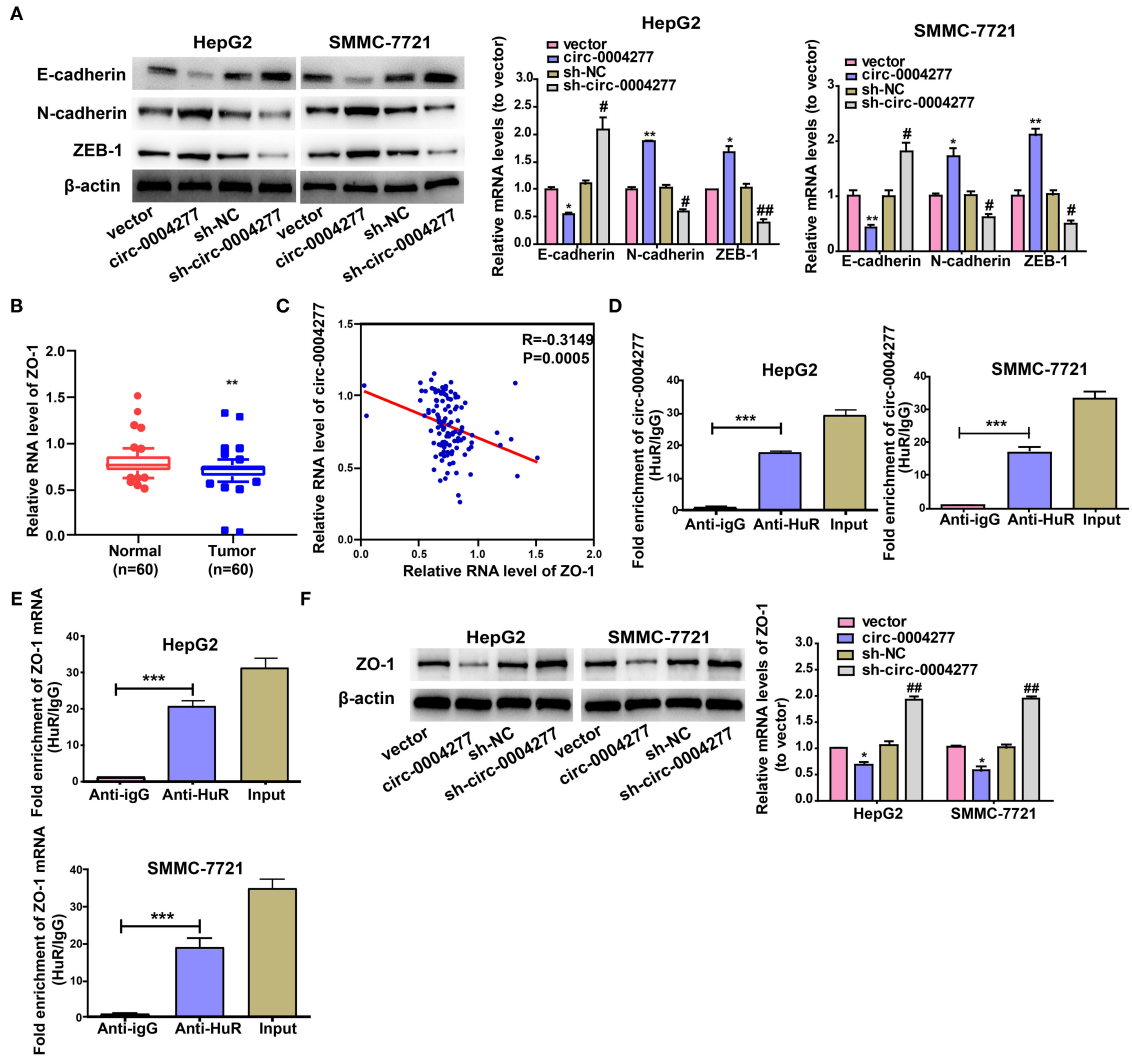
Circ-0004277 promotes EMT progression and inhibits ZO-1. Circ-0004277/NC shRNA lentiviral vector was transfected into HCC cells, namely sh-circ-0004277 and sh-NC, respectively. Circ-0004277/ NC lentiviral vector was transfected into HCC cells, named circ-0004277 and vector, respectively. **(A)** The protein and mRNA levels of epithelial marker, mesenchymal marker and EMT-related transcriptional active factor in HCC cells after transfections. **(B)** qRT-PCR detection of the relative expression of *ZO-1* in paired HCC tumor and normal tissues (*n* = 60). **(C)** Correlation between circ-0004277 and *ZO-1* in HCC samples. **(D)** circ-0004277 was enriched in HuR-binding RNAs compared to the control. **(E)** *ZO-1* mRNA was enriched in HuR-binding RNAs compared to the control. **(F)** The protein and mRNA levels of ZO-1 in HCC cells after transfection. Results are reported as mean ± SD. *P < 0.05, **P < 0.01, ***P < 0.001 vs. control group, ^#^*P* < 0.05, ^*##*^*P* < 0.01 vs. sh-NC group. All experiments were performed in triplicate.

As EMT has a vital role in HCC cell migration, we further explored the effect of circ-0004277 on EMT. Expression of the epithelial marker E-cadherin and the mesenchymal marker N-cadherin were determined. As shown in Figure 4A, circ-0004277 overexpression significantly induced the expression of N-cadherin and ZEB-1, and reduced E-cadherin expression at the protein and RNA levels. Consistently, expression of the EMT-related transcription factor ZEB-1 was upregulated upon overexpression of circ-0004277. It is known that ZO-1 contributes to EMT progression. We wondered whether ZO-1 contributes to the progression of HCC. The results showed significantly lower *ZO-1* expression in HCC tumor tissues than in paired adjacent normal tissues (Figure 4B). Interestingly, we found that *ZO-1* expression was inversely correlated with circ-0004277 expression (Figure 4C). CircRNAs mostly act by competing against miRNAs and RNA binding proteins (RBPs) to regulate their target genes (Holdt et al., 2016; Wang Y. et al., 2017; Zeng et al., 2017; Wang et al., 2019). We performed bioinformatics analysis with circInteractome (https://circinteractome.nia.nih.gov/) and identified that HuR could compete against circ-0004277 (Panda et al., 2018). RIP assay verified that circ-0004277 was enriched in HuR-binding RNAs than in the igG (Figure 4D). Meanwhile, we performed bioinformatics analysis with RPISeq (http://pridb.gdcb.iastate.edu/RPISeq/) and identified that HuR also could compete against *ZO-1* mRNA (≥0.9). RIP assay also showed that *ZO-1* was also enriched in HuR binding RNAs than in the igG (Figure 4E). Furthermore, western blot assays showed that circ-0004277 inhibited ZO-1 expression (Figure 4F). These results suggested that circ-0004277 might exert its inhibitive role by competitively binding HuR, therefore, block the binding between HuR and *ZO-1* mRNA, and subsequently inhibits *ZO-1* and stimulates EMT progression.

### Circ-0004277 Stimulates HCC Growth *in vivo*

**Figure 5:**
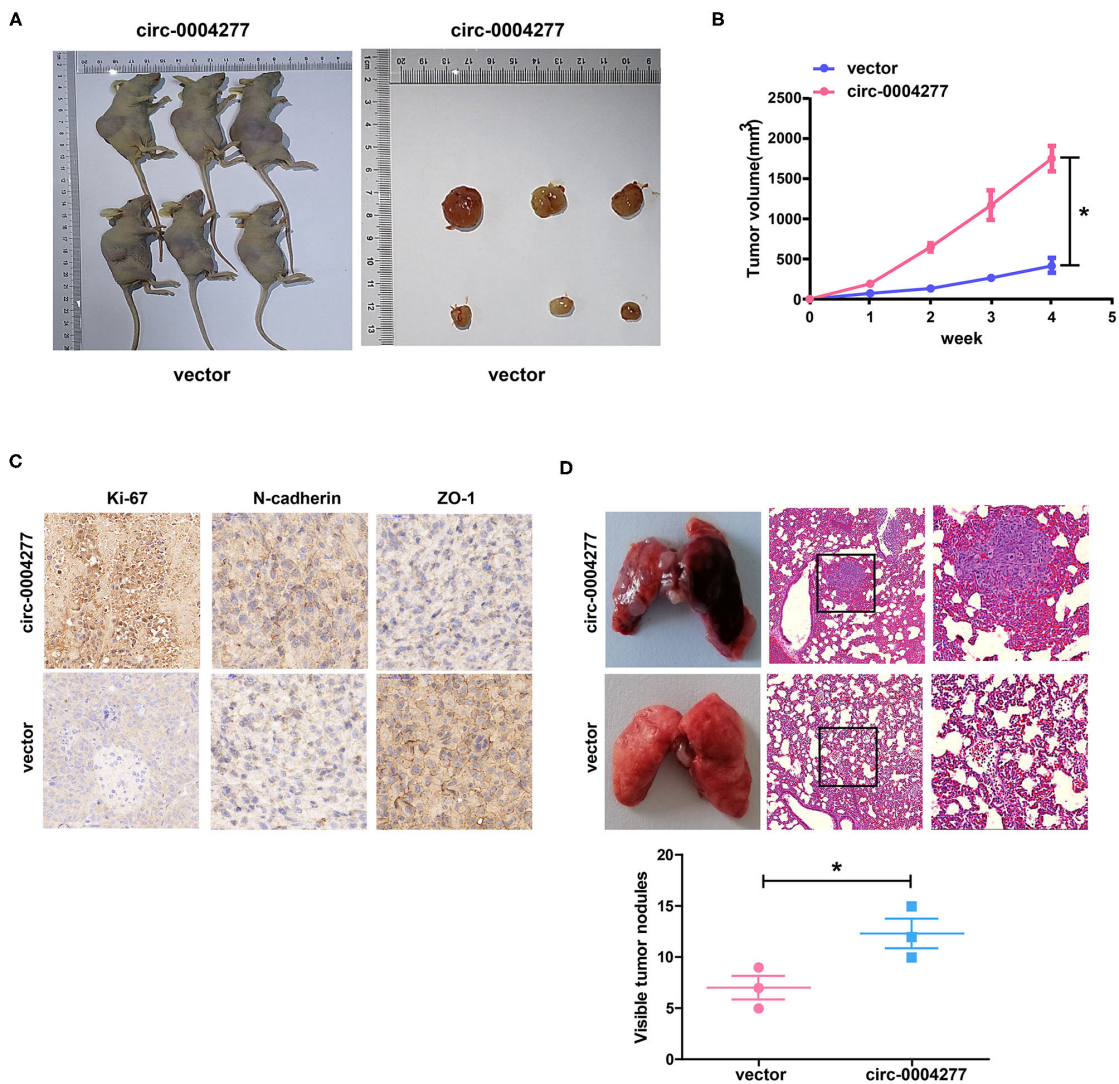
Circ-0004277 stimulates HCC growth *in vivo*. Circ-0004277/ NC lentiviral vector was transfected into SMMC-7721 cells, named circ-0004277 and vector, respectively. **(A)** Representative image of xenografts tumors (*n* = 3 in each group) in nude mice. **(B)** Xenograft tumor volume. **(C)** Immunohistochemical staining of Xenograft tumor in Ki-67, N-cadherin and ZO-1 expression. **(D)** Upregulation of circ-0004277 promotes tumor metastasis *in vivo*. (Left) Representative bright field images of lungs. (Right) Hematoxylin and eosin (HE) staining of lung serial sections. (Bottom) The number of metastatic nodules. Results were reported as mean ± SD. *P < 0.05. All experiments were performed in triplicate.

To study the effect of circ-0004277 on tumor growth *in vivo*, SMMC-7721 cells transfected with circ-0004277-expression or scrambled vectors were subcutaneously injected into nude mice. The results showed that 4 weeks after subcutaneous injection, overexpression of circ-0004277 promoted the formation of nude mice bearing tumors (Figures 5A,B). Immunohistochemical analysis of nude mice bearing tumors showed that overexpression of circ-0004277 inhibited the expression of epithelial marker ZO-1 and promoted the expression of proliferation-specific gene Ki-67 and mesenchymal marker N-cadherin (Figure 5C). Moreover, the results of lung metastasis assays showed that there were significantly more pulmonary metastatic nodules in the mice subcutaneously injected with SMMC-7721 cells transfected with the circ-0004277 overexpression vector than in the control group (Figure 5D). The above results were identical to those of the *in vitro* experiments.

### Exosomal Circ-0004277 Mediates Intercellular Transfer

**Figure 6:**
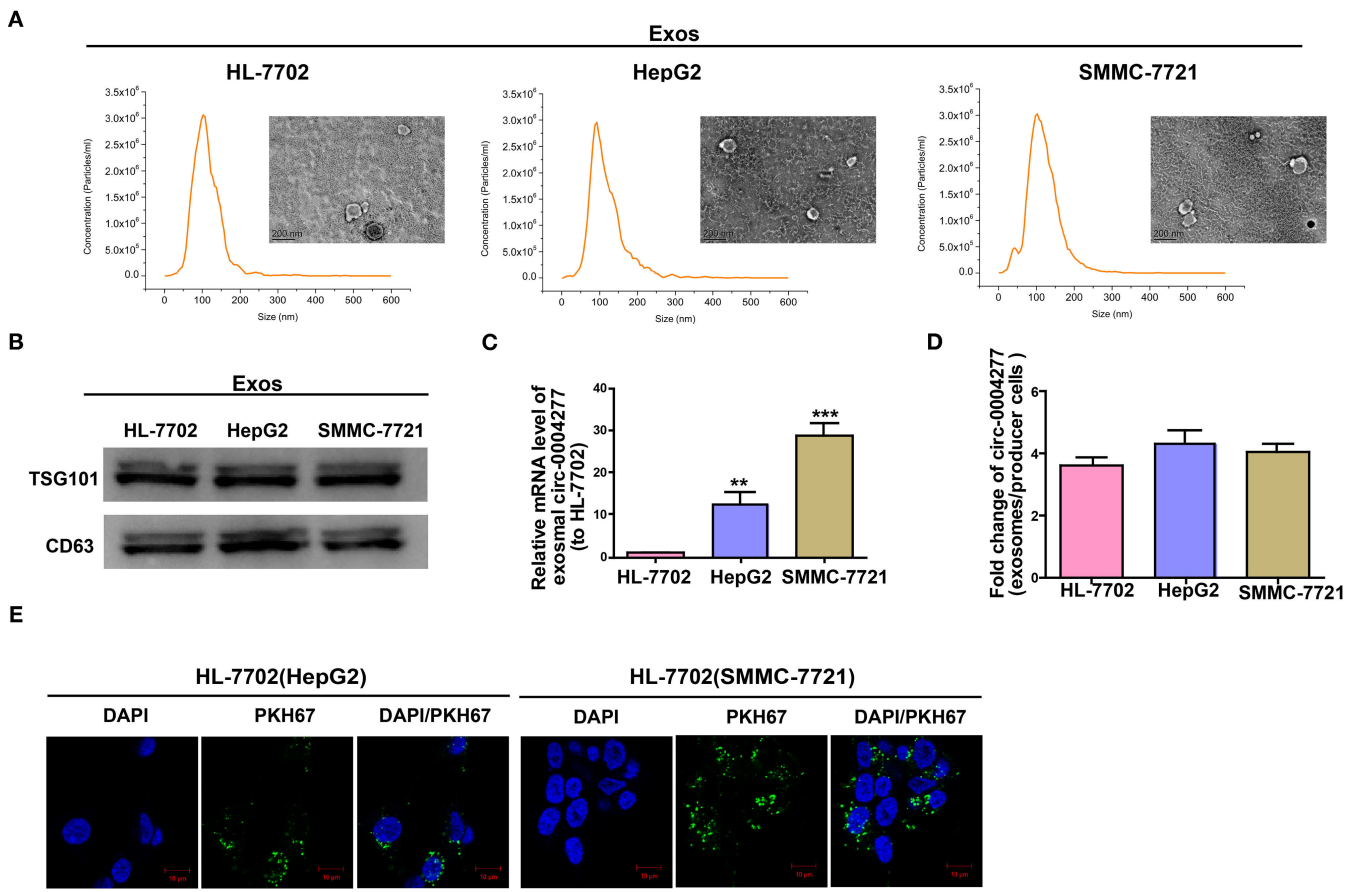
Exosomal circ-0004277 mediates intercellular transfer. Exosomes (Exos) isolated from the medium of HL-7702, SMMC-7721, and HepG2 cells. **(A)** Micrographs of exosomes isolated from HL-7702 (left), HepG2 (middle), and SMMC-7721 cells (right) were detected by TEM (bars = 200 nm) and their size distribution were measured using NTA. **(B)** Western blot analysis of TSG101 and CD63 in exosomes of cell lines. **(C)** The levels of exosomal circ-0004277 in SMMC-7721, HepG2, and HL-7702 cells. **(D)** qRT-PCR detection of the fold change of circ-0004277 between exos of SMMC-7721, HepG2 and HL-7702 and their producer cells. **(E)** Exos of SMMC-7721 and HepG2 cells were labeled with PKH67; green represents PKH67, and blue represents nuclear DNA staining by DAPI. HL-7702 cells were incubated with exosomes derived from SMMC-7721 and HepG2 cells for 3 h. Results are reported as mean ± SD. **P < 0.01, ***P < 0.001. All experiments were performed in triplicate.

We extracted exosomes from HL-7702 and HCC cells, confirmed their size using TEM and NTA (Figure 6A), and conducted western blotting analysis to determine the existence of CD63 and TSG101 (Figure 6B). As expected, HL-7702 cells showed significantly decreased exosomal circ-0004277 levels compared to the levels in HepG2 and SMMC-7721 cells (Figure 6C). Moreover, circ-0004277 levels in exosomes were enriched four times relative to producer cells (Figure 6D). Therefore, exosomes from SMMC-7721 and HepG2 cells contained more circ-0004277 than those from HL-7702 cells. The green fluorescent marker PKH67 was then used to label the exosomes from SMMC-7721 and HepG2 cells. Surprisingly, PKH67 was located in the cytoplasm of recipient cells 3 h after incubation of recipient cells (HL-7702 cells) and labeled exosomes from HepG2 and SMMC-7721 cells (Figure 6E). This indicates that HepG2 and SMMC-7721 cells delivered circ-0004277 into peripheral cells via exosome secretion.

### Effect of Exosomal Circ-0004277 on Normal Cells

**Figure 7:**
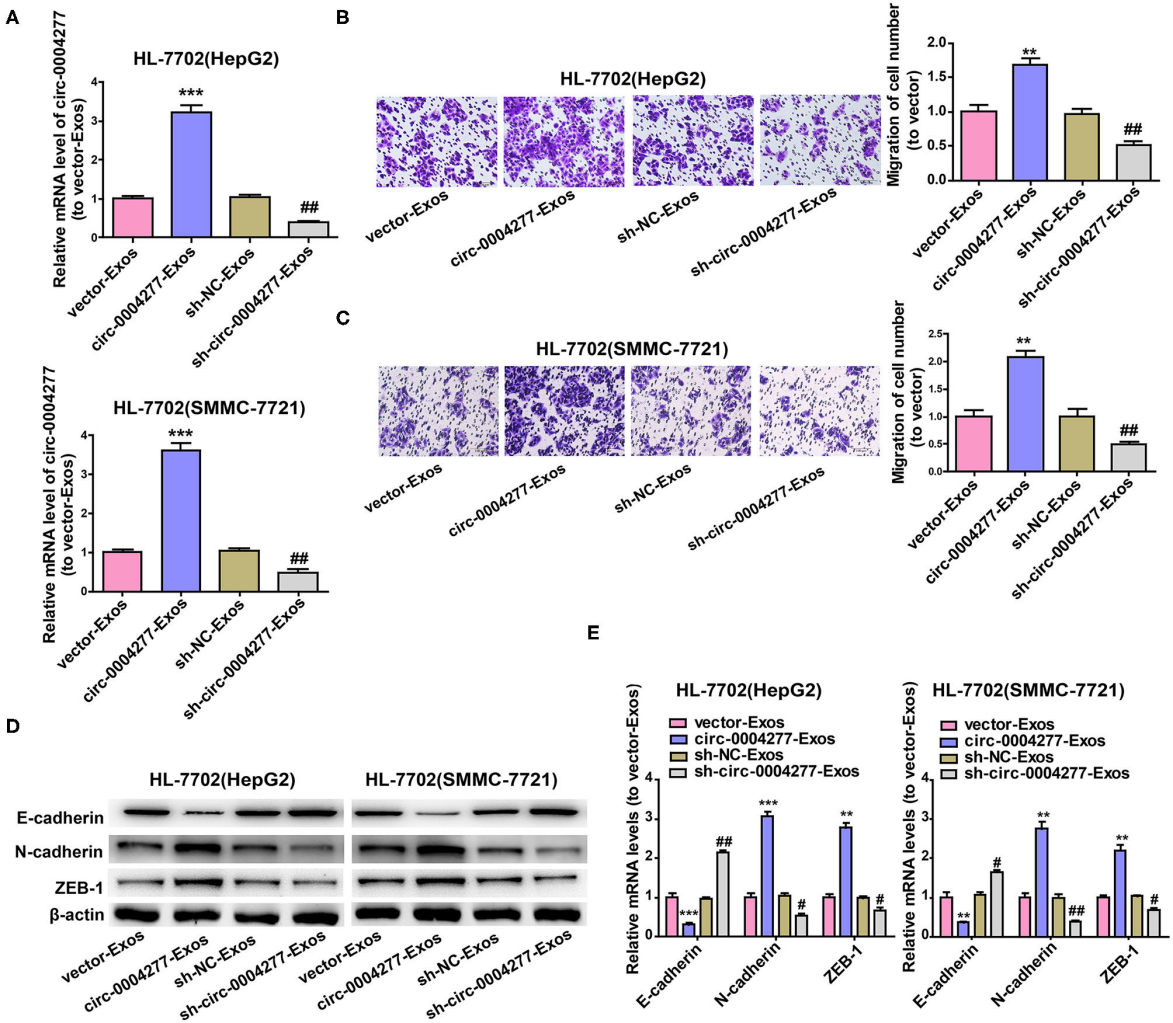
Effect of exosomal circ-0004277 on normal cells. Exosomes (Exos) were isolated from HCC cells transfected with circ-0004277-expressing lentiviral vector, NC lentiviral vector, circ-0004277 shRNA lentiviral vector or NC shRNA lentiviral vector named circ-0004277-Exos, vector-Exos, sh-circ-0004277-Exos and sh-NC-Exos, respectively. Their exosomes were extracted and added to HL-7702 cells for 24 h. **(A)** After transfection with exosomes, qRT-PCR detection of the circ-0004277 levels in HL-7702 cells. **(B)** Representative images of cell migration assays of HL-7702 cells after transfection with exosomes from HepG2. The number of cells were counted. **(C)** Representative images of cell migration assays of HL-7702 cells after transfection with exosomes from SMMC-7721. The number of cells were counted. **(D,E)** The protein **(D)** and mRNA **(E)** levels of epithelial marker, mesenchymal marker and EMT-related transcriptional active factor in HL-7702 cells after cocultivation of HL-7702 cells and Exos form HepG2 or SMMC-7721 cells for 2 weeks. Results are presented as mean ± SD. **P < 0.01, ***P < 0.001 vs. control group, ^#^*P* < 0.05, ^*##*^*P* < 0.01 vs. sh-NC-Exos group. All of the experiments were performed in triplicate.

**Figure 8:**
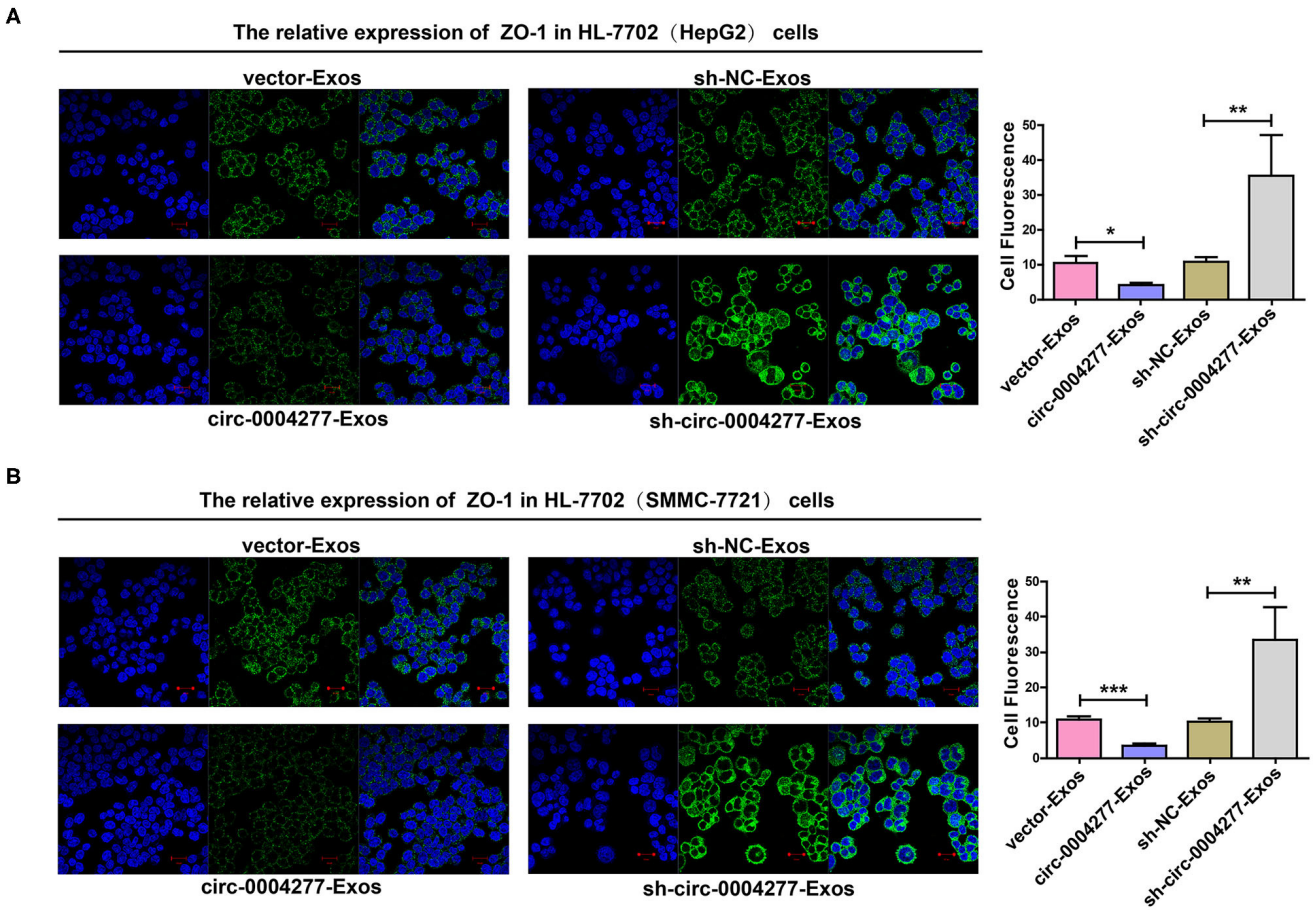
Effect of exosomal circ-0004277 on the expression of ZO-1 in normal cells. Exosomes (Exos) were isolated from HCC cells transfected with circ-0004277-expressing lentiviral vector, NC lentiviral vector, circ-0004277 shRNA lentiviral vector or NC shRNA lentiviral vector namely circ-0004277-Exos, vector-Exos, sh-circ-0004277-Exos and sh-NC-Exos, respectively. Their exosomes were extracted and added to the HL-7702 cells for 24 h. **(A)** Representative images of cell immunofluorescence of ZO-1 in HL-7702 cells after transfection with exosomes from HepG2. The fluorescence of cells was analyses by Image J. **(B)** Representative images of cell immunofluorescence of ZO-1 in HL-7702 cells after transfection with exosomes from SMMC-7721. The fluorescence of cells was analyses by Image J. Results are reported as mean ± SD. *P < 0.05, **P < 0.01, ***P < 0.001. All experiments were performed in triplicate.

Exosomes are important in intercellular communication (Wesolowska and Piwocka, 2017). As described in this study, circ-0004277 was transferred from HepG2 and SMMC-7721 cells into HL-7702 cells in the form of exosomes. Thus, we assumed that exosomal circ-0004277 from SMMC-7721 and HepG2 cells changed the biological function of HL-7702 cells. To determine the function of exosomal circ-0004277, exosomes isolated from HCC cells after transfection with circ-0004277/NC shRNA lentiviral vectors were named sh-circ-0004277-Exos or sh-NC-Exos respectively. Meanwhile, exosomes isolated from HCC cells after transfection with circ-0004277-expression/NC lentiviral vectors were named circ-0004277-Exos or vector-Exos respectively. 100 μg/ml exosomes were added into HL-7702 cells for 24 h. The results indicated that circ-0004277 levels were considerably increased in HL-7702 cells transfected with circ-0004277-Exos and reduced in cells transfected with sh-circ-0004277-Exos (Figure 7A). In addition, sh-circ-0004277-Exos significantly reduced the migration of HL-7702 cells (Figures 7B,C). As expected, circ-0004277-Exos stimulated the migration of HL-7702 cells (Figures 7B,C). Since EMT is crucial in the migration of HCC cells, the effect of circ-0004277-Exos on epithelial features was further explored after 2 weeks cocultivation. We detected the expression of the mesenchymal marker N-cadherin and the epithelial marker E-cadherin. N-cadherin expression was significantly induced, and E-cadherin expression was reduced at the protein and mRNA levels in HL-7702 cells transfected with circ-0004277-Exos. The expression of EMT-related transcription factor ZEB-1 was consistently upregulated during transfection with circ-0004277-Exos (Figures 7D,E). Meanwhile, it was found that sh-circ-0004277-Exos had the opposite effect (Figures 7D,E). Therefore, we wondered whether exosomal circ-0004277 affects the expression of ZO-1 in normal cells. As expected, the results showed that circ-0004277-Exos inhibited the expression of ZO-1 and sh-circ-0004277-Exos had the opposite function (Figures 8A,B). In conclusion, upregulated circ-0004277 stimulated the malignant phenotype of HCC cell lines. In addition, circ-0004277 delivered by exosomes stimulated the EMT process of normal surrounding cells to further promote the progression of HCC into normal surrounding tissues.

## Discussion

As a major etiological factor of cancer-related deaths worldwide, the survival rate of patients with hepatocellular carcinoma remains unsatisfactory, although many efforts have been made to provide better treatment (Asai et al., 2017; Wang T. et al., 2017). With the increase in HCC incidence yearly, seeking a method to facilitate early diagnosis and inhibition or blockage of the migration of HCC cells has become an important task. Recent studies found that circRNA regulated and controlled gene transcription as a target for disease diagnosis. For instance, Shang X et al. found that hsa_circ_0005075 may act as a biomarker for HCC (Shang et al., 2016). Thus, circRNA is closely associated with the occurrence of HCC.

Six well-known circRNAs (circ-0000284, circ-0004277, circ-Cdr1as, circ-Foxo3, circ-SHPRH, and circ-ITCH) were selected in this study to detect their expression in HCC cells through qRT-PCR. These circRNAs are all tumor-related. For example, circ-Cdr1as is related to osteosarcoma (Xu et al., 2018), laryngeal squamous cell carcinoma (Zhang J. et al., 2018) lung cancer (Zhang X. et al., 2018), and hepatocellular carcinoma (Yang et al., 2017). The results showed significantly higher circ-0004277 expression in HCC cell lines relative to normal HL-7702 cells. Circ-0004277 also showed higher expression in HCC tumors and plasma exosomes. Subsequent ROC analysis verified that circ-0004277 is a diagnostic biomarker for HCC detection in clinical practice. *In vitro* and *in vivo* experiments showed that upregulated expression of circ-0004277 significantly promoted cell proliferation and migration, indicating that circ-0004277 is an important positive regulator of HCC cell growth and acts as an oncogene. Therefore, an in-depth study of the mechanisms of circ-0004277 promoting HCC cell growth is very important for understanding the occurrence and development of HCC. CircRNAs can act by competing against RNA binding proteins (RBPs) to regulate their target genes (Holdt et al., 2016; Zeng et al., 2017). Our results showed that circ-0004277 and *ZO-1* mRNA could absorb the same RBP HuR. Further assays suggested that circ-0004277 might exert its inhibitive role by competitively binding HuR, therefore, block the binding between HuR and *ZO-1* mRNA, and subsequently inhibits *ZO-1* and stimulates EMT progression. Nevertheless, the molecular hypotheses still need more evidence to prove it. We would further verify it in our subsequent study.

The level of steady-state circRNA expression in cells can be regulated at three levels (Li X. et al., 2018). First, regulation of circRNA biogenesis is initiated by and coupled with the transcription of circRNA-producing pre-mRNA by Pol II. Second, cis and trans regulatory factors can further influence the efficiency of back-splicing, which is catalyzed by spliceosomal machineries. These factors include ICSs flanking circle formation exons, core spliceosomal components, and other regulatory RBPs. Third, circRNA turnover also plays a role in their expression levels. We will further explore whether circ-0004227 is regulated at these three levels in future studies.

In the present study, circ-0004277 levels were found to be increased in HCC cells and corresponding exosomes and that circ-0004277 expression was approximately four times higher in exosomes than in producer cells. These results indicated that circ-0004277 was mainly located in exosomes. Many studies to date have indicated that exosomal circRNAs reflect the physiological status of donor cells and induce a series of cell reactions after they are captured by recipient cells (O'leary et al., 2017; Zhang et al., 2018a,b). TEM analysis revealed the shape and size of exosomes from HCC and the normal control cell line HL-7702. We further verified exosomes by detecting the exosome markers TSG101 and CD63 (Ha et al., 2016; Khushman et al., 2017). Similar to other reports (Huang A. et al., 2017; Koh et al., 2018), fluorescence microscopy revealed that PKH67 labeled exosomes from HCC cells may migrate into HL-7702 cells. These findings support the possibility that HCC cells may deliver circ-0004277 to HL-7702 cells via exosome secretion. Our study results indicated that circ-0004277-Exos from HCC cells enhanced circ-0004277 expression in HL-7702 cells, stimulated the proliferation and migration of HL-7702 cells and promoted the EMT process. Furthermore, circ-0004277-Exos from HCC cells reduced the level of *ZO-1*. Nevertheless, more mechanistic studies need be performed to further verified our conclusion. In conclusion, our study indicated that circ-0004277 may be transferred into normal surrounding cells directly from HCC cells via exosomes and regulated the biological function of normal surrounding cells via inhibition of ZO-1 and promotion of EMT progression. Understanding the mechanisms by which circ-0004277 promotes the proliferation and migration of HCC cells via inhibition of ZO-1 and promotes EMT progression will be essential to developing novel clinical therapies to prevent and control HCC.

## Data Availability Statement

The raw data supporting the conclusions of this article will be made available by the authors, without undue reservation.

## Ethics Statement

The studies involving human and animal participants were reviewed and approved by the Ethics Committee of the Affiliated Huaian No.1 People's Hospital of Nanjing Medical University. The patients/participants provided their written informed consent to participate in this study.

## Author Contributions

JW and CZ designed the study. CZ and YS performed the experiments. YS and LL wrote the manuscript. SW and YL collected the samples. All authors contributed to the writing and reviewing of the manuscript, and approved the final manuscript for submission.

## Conflict of Interest

The authors declare that the research was conducted in the absence of any commercial or financial relationships that could be construed as a potential conflict of interest.

## References

[B1] Asai A. Tsuchimoto Y. Ohama H. Fukunishi S. Tsuda Y. Kobayashi M.. (2017). Host antitumor resistance improved by the macrophage polarization in a chimera model of patients with HCC. Oncoimmunology 6:e1299301. 10.1080/2162402X.2017.1299301 28507807 PMC5414886

[B2] Basler K. Bergmann S. Heisig M. Naegel A. Zorn-Kruppa M. Brandner J. M. (2016). The role of tight junctions in skin barrier function and dermal absorption. J. Control Release 242, 105–118. 10.1016/j.jconrel.2016.08.007 27521894

[B3] Bosetti C. Bianchi C. Negri E. Colombo M. La Vecchia C. (2009). Estimates of the incidence and prevalence of hepatocellular carcinoma in Italy in 2002 and projections for the years 2007 and 2012. Tumori 95, 23–27. 10.1177/030089160909500104 19366051

[B4] Bruix J. Sherman M. American Association for the Study of Liver Disease. (2011). Management of hepatocellular carcinoma: an update. Hepatology 53, 1020–1022. 10.1002/hep.24199 21374666 PMC3084991

[B5] Carr B. I. Guerra V. (2017). Validation of a liver index and its significance for HCC aggressiveness. J. Gastrointest Cancer 48, 262–266. 10.1007/s12029-017-9971-4 28631027

[B6] Eger N. Schoppe L. Schuster S. Laufs U. Boeckel J. N. (2018). Circular RNA splicing. Adv. Exp. Med. Biol. 1087, 41–52. 10.1007/978-981-13-1426-1_4 30259356

[B7] Forner A. Llovet J. M. Bruix J. (2012). Hepatocellular carcinoma. Lancet 379, 1245–1255. 10.1016/S0140-6736(11)61347-0 22353262 PMC13537732

[B8] Gong Y. Mao J. Wu D. Wang X. Li L. Zhu L.. (2018). Circ-ZEB1.33 promotes the proliferation of human HCC by sponging miR-200a-3p and upregulating CDK6. Cancer Cell. Int. 18:116. 10.1186/s12935-018-0602-3 30123094 PMC6090603

[B9] Ha D. Yang N. Nadithe V. (2016). Exosomes as therapeutic drug carriers and delivery vehicles across biological membranes: current perspectives and future challenges. Acta Pharm. Sin. B 6, 287–296. 10.1016/j.apsb.2016.02.001 27471669 PMC4951582

[B10] Haas A. J. Zihni C. Ruppel A. Hartmann C. Ebnet K. Tada M.. (2020). Interplay between extracellular matrix stiffness and JAM-A regulates mechanical load on ZO-1 and tight junction assembly. Cell Rep. 32:107924. 10.1016/j.celrep.2020.107924 32697990 PMC7383227

[B11] Han D. Li J. Wang H. Su X. Hou J. Gu Y.. (2017). Circular RNA circMTO1 acts as the sponge of microRNA-9 to suppress hepatocellular carcinoma progression. Hepatology 66, 1151–1164. 10.1002/hep.29270 28520103

[B12] Henri O. Pouehe C. Houssari M. Galas L. Nicol L. Edwards-Levy F.. (2016). Selective stimulation of cardiac lymphangiogenesis reduces myocardial edema and fibrosis leading to improved cardiac function following myocardial infarction. Circulation 133, 1484–1497. 10.1161/CIRCULATIONAHA.115.020143 26933083

[B13] Holdt L. M. Stahringer A. Sass K. Pichler G. Kulak N. A. Wilfert W.. (2016). Circular non-coding RNA ANRIL modulates ribosomal RNA maturation and atherosclerosis in humans. Nat. Commun. 7:12429. 10.1038/ncomms12429 27539542 PMC4992165

[B14] Hsiao K. Y. Lin Y. C. Gupta S. K. Chang N. Yen L. Sun H. S.. (2017). Noncoding effects of circular RNA CCDC66 promote colon cancer growth and metastasis. Cancer Res. 77, 2339–2350. 10.1158/0008-5472.CAN-16-1883 28249903 PMC5910173

[B15] Hsu Y. L. Hung J. Y. Chang W. A. Lin Y. S. Pan Y. C. Tsai P. H.. (2017). Hypoxic lung cancer-secreted exosomal miR-23a increased angiogenesis and vascular permeability by targeting prolyl hydroxylase and tight junction protein ZO-1. Oncogene 36, 4929–4942. 10.1038/onc.2017.105 28436951

[B16] Huang A. Isobe N. Yoshimura Y. (2017). Changes in localization and density of CD63-positive exosome-like substances in the hen oviduct with artificial insemination and their effect on sperm viability. Theriogenology 101, 135–143. 10.1016/j.theriogenology.2017.06.028 28708510

[B17] Huang X. Y. Huang Z. L. Xu Y. H. Zheng Q. Chen Z. Song W.. (2017). Comprehensive circular RNA profiling reveals the regulatory role of the circRNA-100338/miR-141-3p pathway in hepatitis B-related hepatocellular carcinoma. Sci. Rep. 7:5428. 10.1038/s41598-017-05432-8 28710406 PMC5511135

[B18] Jin X. Feng C. Y. Xiang Z. Chen Y. P. Li Y. M. (2016). CircRNA expression pattern and circRNA-miRNA-mRNA network in the pathogenesis of nonalcoholic steatohepatitis. Oncotarget 7, 66455–66467. 10.18632/oncotarget.12186 27677588 PMC5341813

[B19] Khushman M. Bhardwaj A. Patel G. K. Laurini J. A. Roveda K. Tan M. C.. (2017). Exosomal markers (CD63 and CD9) expression pattern using immunohistochemistry in resected malignant and nonmalignant pancreatic specimens. Pancreas 46, 782–788. 10.1097/MPA.0000000000000847 28609367 PMC5494969

[B20] Kim D. W. Talati C. Kim R. (2017). Hepatocellular carcinoma (HCC): beyond sorafenib-chemotherapy. J. Gastrointest. Oncol. 8, 256–265. 10.21037/jgo.2016.09.07 28480065 PMC5401857

[B21] Koh Y. Q. Almughlliq F. B. Vaswani K. Peiris H. N. Mitchell M. D. (2018). Exosome enrichment by ultracentrifugation and size exclusion chromatography. Front. Biosci. 23, 865–874. 10.2741/4621 28930577

[B22] Li J. Li Z. Jiang P. Peng M. Zhang X. Chen K.. (2018). Circular RNA IARS (circ-IARS) secreted by pancreatic cancer cells and located within exosomes regulates endothelial monolayer permeability to promote tumor metastasis. J. Exp. Clin. Cancer Res. 37:177. 10.1186/s13046-018-0822-3 30064461 PMC6069563

[B23] Li X. Yang L. Chen L. L. (2018). The biogenesis, functions, and challenges of circular RNAs. Mol. Cell 71, 428–442. 10.1016/j.molcel.2018.06.034 30057200

[B24] Lochhead J. J. Yang J. Ronaldson P. T. Davis T. P. (2020). Structure, function, and regulation of the blood-brain barrier tight junction in central nervous system disorders. Front. Physiol. 11:914. 10.3389/fphys.2020.00914 32848858 PMC7424030

[B25] Ma Y. Pan J. Z. Zhao S. P. Lou Q. Zhu Y. Fang Q. (2016). Microdroplet chain array for cell migration assays. Lab. Chip 16, 4658–4665. 10.1039/C6LC00823B 27833945

[B26] Niendorf E. Spilseth B. Wang X. Taylor A. (2015). Contrast enhanced MRI in the diagnosis of HCC. Diagnostics 5, 383–398. 10.3390/diagnostics5030383 26854161 PMC4665604

[B27] O'leary V. B. Smida J. Matjanovski M. Brockhaus C. Winkler K. Moertl S.. (2017). The circRNA interactome-innovative hallmarks of the intra- and extracellular radiation response. Oncotarget 8, 78397–78409. 10.18632/oncotarget.19228 29108237 PMC5667970

[B28] Pan J. Ding M. Xu K. Yang C. Mao L. J. (2017). Exosomes in diagnosis and therapy of prostate cancer. Oncotarget 8, 97693–97700. 10.18632/oncotarget.18532 29228644 PMC5722596

[B29] Panda A. C. Dudekula D. B. Abdelmohsen K. Gorospe M. (2018). Analysis of circular RNAs using the web tool circinteractome. Methods Mol. Biol. 1724, 43–56. 10.1007/978-1-4939-7562-4_4 29322439 PMC5897125

[B30] Shang X. Li G. Liu H. Li T. Liu J. Zhao Q.. (2016). Comprehensive circular RNA profiling reveals that hsa_circ_0005075, a new circular RNA biomarker, is involved in hepatocellular crcinoma development. Medicine 95:e3811. 10.1097/MD.0000000000003811 27258521 PMC4900729

[B31] Steinbichler T. B. Dudas J. Riechelmann H. Skvortsova I. (2017). The role of exosomes in cancer metastasis. Semin. Cancer Biol. 44, 170–181. 10.1016/j.semcancer.2017.02.006 28215970

[B32] Su Y. Lv X. Yin W. Zhou L. Hu Y. Zhou A.. (2019). CircRNA Cdr1as functions as a competitive endogenous RNA to promote hepatocellular carcinoma progression. Aging 11, 8182–8203. 10.18632/aging.102312 31581132 PMC6814590

[B33] Tian M. Chen R. Li T. Xiao B. (2018). Reduced expression of circRNA hsa_circ_0003159 in gastric cancer and its clinical significance. J. Clin. Lab. Anal. 32:e22281. 10.1002/jcla.22281 28618205 PMC6817154

[B34] Wang S. Hu Y. Lv X. Li B. Gu D. Li Y.. (2019). Circ-0000284 arouses malignant phenotype of cholangiocarcinoma cells and regulates the biological functions of peripheral cells through cellular communication. Clin. Sci. 133, 1935–1953. 10.1042/CS20190589 31501232

[B35] Wang T. Xu L. Jia R. Wei J. (2017). MiR-218 suppresses the metastasis and EMT of HCC cells via targeting SERBP1. Acta Biochim. Biophys. Sin. 49, 383–391. 10.1093/abbs/gmx017 28369267

[B36] Wang Y. Mo Y. Gong Z. Yang X. Yang M. Zhang S. (2017). Circular RNAs in human cancer. Mol. Cancer 16:25 10.1186/s12943-017-0598-7 28143578 PMC5282898

[B37] Wesolowska A. Piwocka K. (2017). [Exosomal microRNAs as a part of the cell-cell communication in cancer]. Postepy Biochem. 63, 110–118. 28689377

[B38] Xu B. Yang T. Wang Z. Zhang Y. Liu S. Shen M. (2018). CircRNA CDR1as/miR-7 signals promote tumor growth of osteosarcoma with a potential therapeutic and diagnostic value. Cancer Manag Res. 10, 4871–4880. 10.2147/CMAR.S178213 30425578 PMC6203089

[B39] Xu L. Zhang M. Zheng X. Yi P. Lan C. Xu M. (2017). The circular RNA ciRS-7 (Cdr1as) acts as a risk factor of hepatic microvascular invasion in hepatocellular carcinoma. J. Cancer Res. Clin. Oncol. 143, 17–27. 10.1007/s00432-016-2256-7 27614453 PMC11819007

[B40] Yang X. Xiong Q. Wu Y. Li S. Ge F. (2017). Quantitative proteomics reveals the regulatory networks of circular RNA CDR1as in hepatocellular carcinoma cells. J. Proteome Res. 16, 3891–3902. 10.1021/acs.jproteome.7b00519 28892615

[B41] Yao J. T. Zhao S. H. Liu Q. P. Lv M. Q. Zhou D. X. Liao Z. J.. (2017). Over-expression of CircRNA_100876 in non-small cell lung cancer and its prognostic value. Pathol. Res. Pract. 213, 453–456. 10.1016/j.prp.2017.02.011 28343871

[B42] Zeng Y. Du W. W. Wu Y. Yang Z. Awan F. M. Li X.. (2017). A circular RNA binds to and activates AKT phosphorylation and nuclear localization reducing apoptosis and enhancing cardiac repair. Theranostics 7, 3842–3855. 10.7150/thno.19764 29109781 PMC5667408

[B43] Zhang H. Deng T. Ge S. Liu Y. Bai M. Zhu K.. (2018a). Exosome circRNA secreted from adipocytes promotes the growth of hepatocellular carcinoma by targeting deubiquitination-related USP7. Oncogene 38, 2844–2859. 10.1038/s41388-018-0619-z 30546088 PMC6484761

[B44] Zhang H. Zhu L. Bai M. Liu Y. Zhan Y. Deng T.. (2018b). Exosomal circRNA derived from gastric tumor promotes white adipose browning by targeting the miR-133/PRDM16 pathway. Int. J. Cancer 144, 2501–2515. 10.1002/ijc.31977 30412280

[B45] Zhang J. Hu H. Zhao Y. Zhao Y. (2018). CDR1as is overexpressed in laryngeal squamous cell carcinoma to promote the tumour's progression via miR-7 signals. Cell Prolif. 51:e12521. 10.1111/cpr.12521 30182381 PMC6528957

[B46] Zhang X. Yang D. Wei Y. (2018). Overexpressed CDR1as functions as an oncogene to promote the tumor progression via miR-7 in non-small-cell lung cancer. Onco. Targets Ther. 11, 3979–3987. 10.2147/OTT.S158316 30022841 PMC6044366

[B47] Zhao J. Li L. Wang Q. Han H. Zhan Q. Xu M. (2017). CircRNA expression profile in early-stage lung adenocarcinoma patients. Cell. Physiol. Biochem. 44, 2138–2146. 10.1159/000485953 29241190

[B48] Zhong Z. Huang M. Lv M. He Y. Duan C. Zhang L.. (2017). Circular RNA MYLK as a competing endogenous RNA promotes bladder cancer progression through modulating VEGFA/VEGFR2 signaling pathway. Cancer Lett. 403, 305–317. 10.1016/j.canlet.2017.06.027 28687357

[B49] Zhu Z. Peng L. Chen G. Jiang W. Shen Z. Du C.. (2017). Mutations of MYH14 are associated to anorectal malformations with recto-perineal fistulas in a small subset of Chinese population. Clin. Genet. 92, 503–509. 10.1111/cge.12993 28191911

