# Supplementary material for: Circular RNA hsa_circ_0004277 Stimulates Malignant Phenotype of Hepatocellular Carcinoma and Epithelial-Mesenchymal Transition of Peripheral Cells
Source: Front Cell Dev Biol. 2021 Jan 12;8:585565. doi: 10.3389/fcell.2020.585565 (PMC7835424; doi:10.3389/fcell.2020.585565)
Supplement: Supplementary file 1 [file Table_1.DOCX]

**Supplementary Table 1. The characteristics of the hepatocellular carcinoma cases and negative controls**

| **Variables** | **Cases** | **Controls** | ***χ^2^*** | ***P* value** |
| --- | --- | --- | --- | --- |
| **Total** | 60 | 60 |  |  |
| **Gender** |  |  | 0.045 | 0.831 |
| Male | 46(76.7%) | 45(75.0%) |  |  |
| Female | 14(23.3%) | 15(25.0%) |  |  |
| **Age (years)** |  |  | 0.133 | 0.715 |
| ≤54 | 29(48.3%) | 31(51.7%) |  |  |
| >54 | 31(51.7%) | 29(48.3%) |  |  |
| **HBV infection** |  |  | 1.234 | 0.267 |
| Absent | 22(36.7%) | 28(46.7%) |  |  |
| Present | 38(63.3%) | 32(53.3%) |  |  |
| **Grade of differentiation** |  |  | - | **-** |
| Low | 24(40.0%) | - |  |  |
| Middle | 21(35.0%) | - |  |  |
| High | 15(25.0%) | - |  |  |
| **Tumor diameter (cm)** |  |  | - | - |
| ≤5 | 28(46.7%) | - |  |  |
| >5 | 32(53.3%) | - |  |  |

Two-sided χ^2^ for all variables between hepatocellular carcinoma cases and controls
