# Supplementary material for: Circular RNA hsa_circ_0004277 Stimulates Malignant Phenotype of Hepatocellular Carcinoma and Epithelial-Mesenchymal Transition of Peripheral Cells
Source: Front Cell Dev Biol. 2021 Jan 12;8:585565. doi: 10.3389/fcell.2020.585565 (PMC7835424; doi:10.3389/fcell.2020.585565)
Supplement: Supplementary file 2 [file Data_Sheet_1.DOCX]

**Supplementary Figure 1**

**
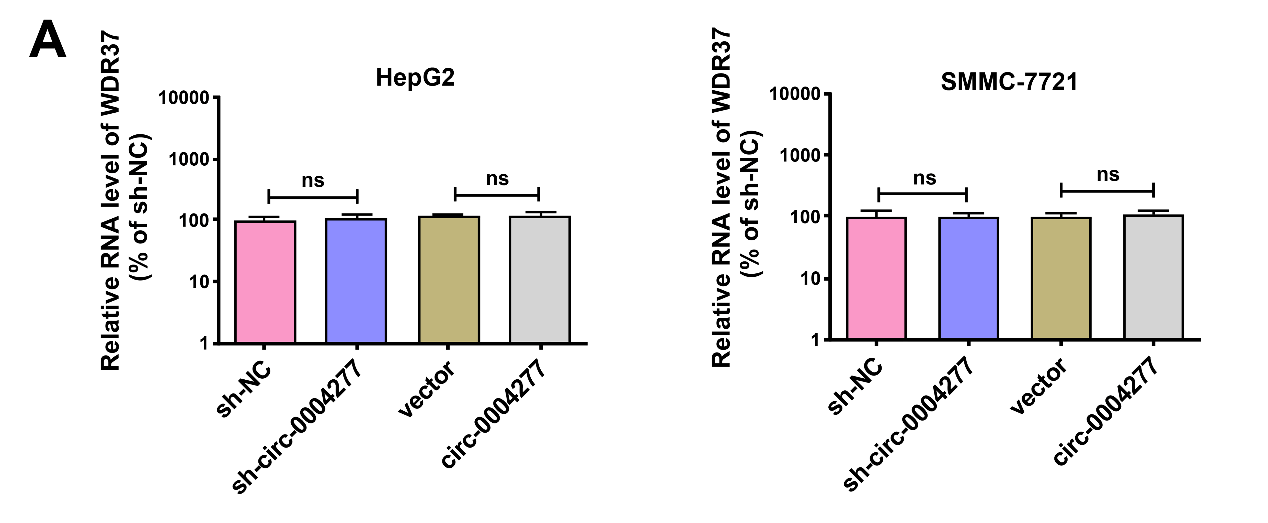
**

**Supplementary Figure 1. The expression of the host gene WDR37 in response to modulation of circ-0004277.** After circ-0004277 overexpression or downregulation, RNA levels of WDR37 in HCC cells were detected by qRT-PCR. Results are reported as mean ± SD. ns: no significance. All experiments were performed in triplicate.
